# Prospective validation of *VEGF* and *eNOS* polymorphisms as predictors of first-line bevacizumab efficacy in patients with metastatic colorectal cancer

**DOI:** 10.1038/s41598-023-40220-7

**Published:** 2023-08-09

**Authors:** Giorgia Marisi, Irene Azzali, Alessandro Passardi, Francesca Rebuzzi, Giulia Bartolini, Milena Urbini, Matteo Canale, Chiara Molinari, Laura Matteucci, Francesco Giulio Sullo, Silvia Angela Debonis, Chiara Gallio, Graziana Gallo, Giovanni Luca Frassineti, Paola Ulivi

**Affiliations:** 1 Biosciences Laboratory, IRCCS Istituto Romagnolo per lo Studio dei Tumori (IRST) “Dino Amadori”, Meldola, Italy; 2 Unit of Biostatistics and Clinical Trials, IRCCS Istituto Romagnolo per lo Studio dei Tumori (IRST) “Dino Amadori”, Meldola, Italy; 3 Department of Medical Oncology, IRCCS Istituto Romagnolo per lo Studio dei Tumori (IRST) “Dino Amadori”, Meldola, Italy; 4 Operative Unit of Pathologic Anatomy, Azienda USL della Romagna, “Bufalini” Hospital, Cesena, Italy

**Keywords:** Molecular biology, Oncology

## Abstract

Bevacizumab (Bev) plus chemotherapy is a standard first-line treatment in metastatic colorectal cancer (mCRC), however to date no predictive factors of response have been identified. Results of our previous analysis on patients enrolled in a randomized prospective phase III multicenter study (ITACa study) showed a predictive value of Vascular Endothelial Growth Factor (*VEGF*) polymorphism (*VEGF* + 936), a 27-nucleotide variable number tandem repeat (VNTR) of the endothelial nitric oxide synthase (*eNOS*) gene and *eNOS* + 894 polymorphism. mCRC patients, treated with Bev plus chemotherapy, were included in this prospective validation trial. *eNOS* + 894G > T was analyzed by Real time PCR, while the *eNOS* VNTR and *VEGF* + 936C > T were determined by standard PCR and direct sequencing analysis. These polymorphisms were assessed in relation to progression-free survival (PFS), overall survival (OS) and objective response rate (ORR). These three polymorphisms were not predictive of PFS (*p* 0.91, 0.59 and 0.09, respectively), and OS (*p* 0.95, 0.32 and 0.46, respectively). Moreover, the haplotype analyses did not confirm what was found in our previous study; patients bearing a specific haplotype of *eNOS* had not significantly improved outcomes. This prospective study failed to validate the predictive impact of *eNOS* and *VEGF* polymorphisms for response to Bev plus first-line chemotherapy in mCRC patients.

## Introduction

Colorectal cancer (CRC) is one of the most common malignancies, globally repre-senting the fourth most diagnosed cancer in the world, third in incidence in men and second in women. CRC is the third leading cause of cancer death in the world, consider-ing both men and women globally^1,2^.

In about 25% of cases, it occurs as a metastatic disease already at diagnosis. In this setting, systemic treatments are considered the gold standard. Associations of fluoropy-rimidines with oxaliplatin and/or irinotecan and a biologic agent are commonly used in the first-line setting. Bevacizumab (Bev), a humanised monoclonal antibody directed against the Vascular Endothelial Growth Factor (VEGF), was the first drug with a molec-ular target established in the first-line treatment of mCRC^3^. A recent meta-analysis showed no benefit in overall survival (OS) from Bev-containing regimens, while adding Bev to chemotherapy significantly improved progression free survival (PFS)^3^. Despite efforts in clinical and translational research covering more than ten years, no biomarkers of response or resistance to Bev treatment have been found to date. Therefore, Bev is com-monly used in association with any chemotherapy regimen and independently of any se-lection factor.

VEGF represents the main pro-angiogenic factor, and acts binding to specific recep-tors present on the membrane of endothelial cells, VEGFR-1 and VEGFR-2. This led to the variation of the structure of the cytoskeleton of the endothelial cells, with the consequent modification of the cell motility. VEGF expression is related to blood vessel formation and metastasization in various cancers.

Several researchers assessed the role of *VEGF* Single Nucleotide Polymorphisms (SNPs) in relation to Bev responsiveness with contradictory findings^4–7^. Our team analyzed *VEGF* and endothelial nitric oxide synthase (*eNOS*) SNPs in relation to clinical out-come (PFS, OS and overall response rate, ORR) in mCRC patients undergoing Bev-based first-line chemotherapy in the phase III prospective multicenter randomized “Italian Trial in Advanced Colorectal Cancer (ITACa)” study^8^. This study demonstrated that *VEGF* 936C/T, *eNOS* + 894 G/T, and VNTR were linked with prognosis only in Bev plus first-line chemotherapy patients. Moreover, patients with a particular haplotype combination of the two *eNOS* polymorphisms (defined as *eNOS* Haplo1/Haplo1 and *eNOS* Haplo 2/Haplo2), in comparison to those with the other genotypes, demonstrated significantly longer PFS and OS, as well as a higher ORR^8^.

The results of the abovementioned work needed to be confirmed in an independent case series, thus the present trial was designed to validate the role of these polymorphisms and haplotypes. If confirmed, these data could represent a valid selection criterion for candidates for treatment with Bev.

## Results

From January 2016 to October 2019, 182 patients were enrolled into this trial. Thirteen patients were excluded from the analysis due to eligibility criteria violation. Table 1 summarizes the patient characteristics. Fifty-five percent of patients were men and the median age was 69 years. ECOG PS was equal to 0 in 75% of cases, 1 in 23% of cases and equal to 2 in a minority (2%). As regards the localization of the primary tumor, 69 patients (42%) had a neoplasm originating from the left colon, 64 patients (39%) from the right colon and 32 patients (19%) from the rectum. Analyzing the site of metastases, 58% of patients had me-tastases to the liver, 37% to the lungs and 24% to the peritoneum. Grade 3 adenocarcinomas were the most represented (38%). Prior surgery for primary tumor and adjuvant therapy occurred in 74% and 30% of cases, respectively. Most patients (88%) received a first-line chemotherapy treatment containing intravenous fluoropyrimidine and oxaliplatin or irinotecan (specifically, 107 patients received FOLFOX and 41 FOLFIRI). As regards mutational analyses performed on tissue, *KRAS*, *NRAS* e *BRAF* mutations were detected in 58%, 7% and 13% of cases, respectively. Table 2 shows the distribution of the polymorphisms *VEGF* + 936, *eNOS* + 894G > T and *eNOS* VNTR in the study population. At a median follow-up of 52.6 months, there had been 161 (95%) progressions and 134 (79%) deaths in the cohort under analysis. Median PFS and OS were 12.2 months (95% CI 11.2–13.4) and 22.6 months (95% 18.6–29.1), respectively. During treatment, 83 patients (72%) reached a CR or PR as best overall response.

**Table 1:** Patient characteristics.

Variable	Overall n = 169 (%)
Age	
Median (range)	69 (33–85)
Gender	
Female	76 (45%)
Male	93 (55%)
Grading	
1	14 (9%)
2	53 (34%)
3	59 (38%)
4	30 (19%)
Unknown	13
ECOG PS	
0	127 (75%)
1	39 (23%)
2	3 (2%)
Tumor localization	
Rectum	32 (19%)
Right colon	64 (39%)
Left colon	69 (42%)
Unknown	4
Site of metastases	
Liver	98 (58%)
Lung	63 (37%)
Peritoneum	40 (24%)
Metastases	
Sinchronous	79 (52%)
Metachronous	74 (48%)
Missing	16
Stage at diagnosis in metachronous group	
Stage I	3 (5%)
Stage II	14 (33%)
Stage III	26 (60%)
Missing	31
Chemotherapy regimen	
FOLFOX	107 (63%)
FOLFIRI	41 (24%)
CAPOX	12 (7%)
CAPIRI	1 (1%)
Other	8 (5%)
Previous surgery	
Yes	124 (74%)
No	44 (26%)
Unknown	1
Previous adjuvant chemotherapy	
Yes	50 (30%)
No	117 (70%)
Unknown	2
*KRAS*	
Wild type	67 (42%)
Mutated	93 (58%)
Unknown	9
*NRAS*	
Wild type	141 (93%)
Mutated	10 (7%)
Unknown	18
*BRAF*	
Wild type	122 (87%)
Mutated	19 (13%)
Unknown	28

ECOG PS, Eastern Cooperative Oncology Group Performance Status.

**Table 2:** Genotype frequencies.

	No. of patients (%)
*eNOS* + 894	
GT	84 (54%)
GG	60 (38%)
TT	13 (8%)
Unknown	12
*eNOS* VNTR	
4bb	121 (74%)
4ab	34 (21%)
4aa	9 (5%)
Unknown	5
*VEGF* + 936	
CC	121 (72%)
CT	43 (26%)
TT	3 (2%)
Unknown	2

### Association between polymorphisms and clinical outcomes

We evaluated the correlation between PFS and *VEGF* + 936, *eNOS* + 894 and *eNOS* VNTR polymorphisms. The analysis carried out did not confirm what was found in the previous study, as none of the polymorphisms correlated significantly with PFS (Table 3, Supplementary Fig. S1).

**Table 3:** PFS and OS in relation to polymorphisms.

	Progression-free survival			Overall survival		
	Median PFS (95%CI)	HR (95% CI)	*p*	Median OS (95%CI)	HR (95% CI)	*p*
*eNOS* + 894G						
GT	11.7 (9.86–15.3)	0.95 (0.69–1.32)	0.78	23.4 (17.3–32.3)	0.92 (0.64–1.31)	
GG/TT	12.4 (10.94–13.9)	1.00		24.6 (17.0–32.4)	1.00	0.63
*eNOS* VNTR						
4bb	12.4 (11.27–14.7)	0.74 (0.52–1.06)	0.1	24.3 (19.7–32.1)	0.88 (0.59–1.30)	
4ab/4aa	11.5 (8.74–13.8)	1.00		17.9 (13.7–32.4)	1.00	0.52
*VEGF* + 936						
TT	10.4 (7.36-NA)	0.88 (0.28–2.77)	0.83	20.9 (15.2-NA)	0.95 (0.23–3.86)	
CT/CC	12.4 (11.37–13.7)	1.00		22.6 (18.9–29.8)	1.00	0.94

With regard to OS, no statistically significant difference was observed between polymorphism genotypes (Table 3, Supplementary Fig. S1).

We also investigated *eNOS* + 894 and *eNOS* VNTR polymorphisms in relation to ORR. *VEGF* + 936 was not considered in this analysis due to the small frequencies of CT and TT genotypes. *eNOS* VNTR was the only polymorphism significantly associated with ORR in univariate analysis, but this result was not confirmed in multivariate analysis (Supplemetary Table S1).

### *eNOS* haplotype

In our previous analysis, two haplotypes based on *eNOS* VNTR 4a/b and *eNOS* + 894 G/T were described: *eNOS* Haplo 1 (4b-G), *eNOS* Haplo 2 (4b-T).

Homozygosity in *eNOS* Haplo1 (Haplo1/Haplo1), in *eNOS* Haplo2 (Haplo2/Haplo2) and their combination (Haplo1/Haplo1 + Haplo2/Haplo2) were considered for correlation with patient outcomes.

**Figure 1:**
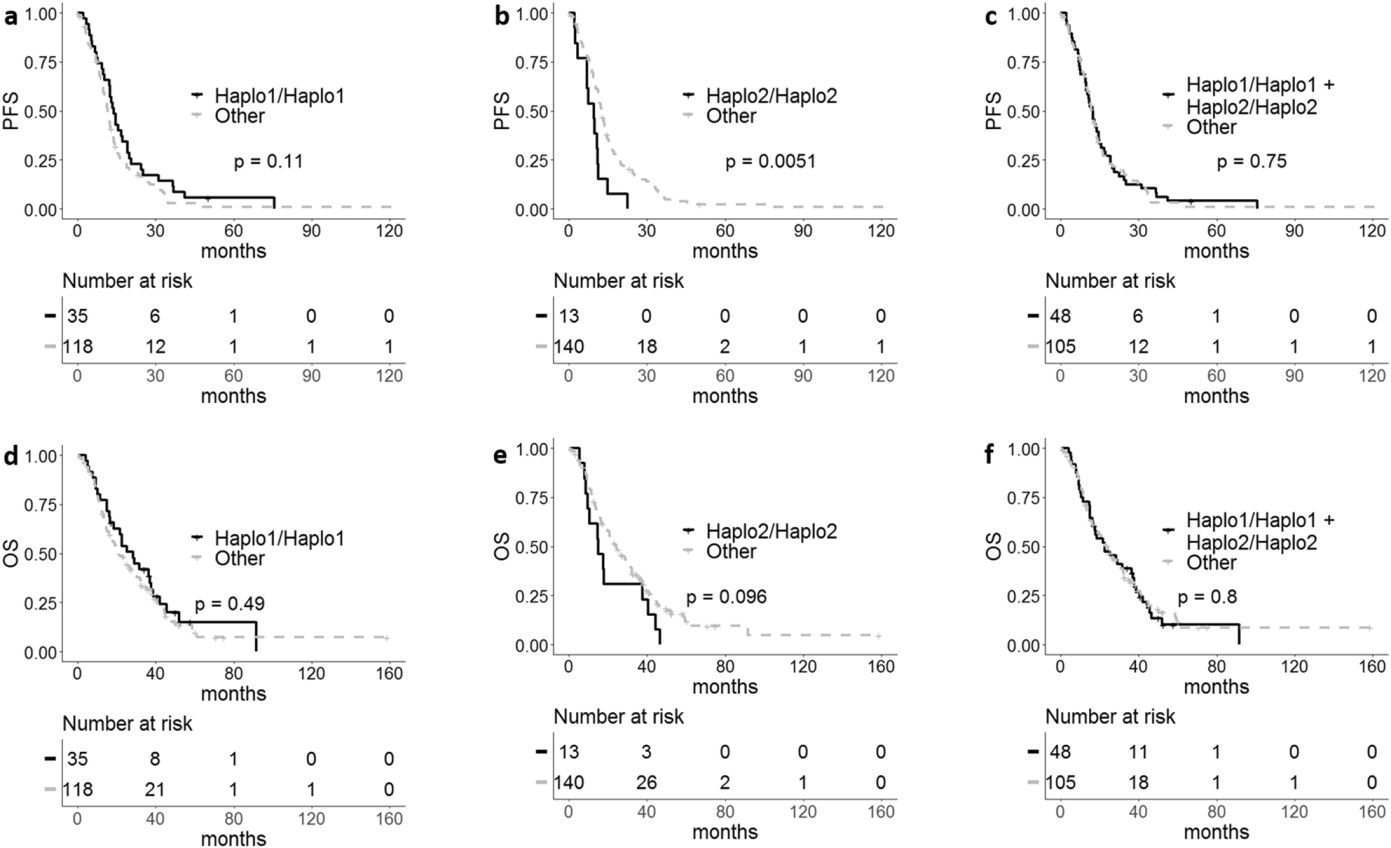
Kaplan Meir curves for *eNOS* haplotypes. (**a**,** b**, **c**) Progression-free survival (PFS) and (**d**, **e**, **f**) overall survival (OS) in relation to Haplo1/Haplo1, Haplo2/Haplo2 or Haplo1/Haplo1 plus Haplo2/Haplo2 genotypes, respectively.

The analyses performed did not confirm what was found in the previous study, in particular patients bearing *eNOS* Haplo1/Haplo1 or the combination of *eNOS* Haplo1/Haplo1 and *eNOS* Haplo2/Haplo2 had not significantly improved outcomes in terms of PFS, OS and ORR (Table 4, Fig. 1, Supplementary Table S2). A significant effect on PFS was instead observed in the group *eNOS* Haplo2/Haplo2. In particular *eNOS* Haplo2/Haplo2 showed worse PFS than other geno-types (HR 2.25, 95% CI 1.26–4.03, *p* = 0.006). The result obtained was confirmed in multivariate analysis (adjusted-HR 2.83, 95% CI 1.33–5.99, *p* = 0.006) (Table 4 and Fig. 1).

**Table 4:** PFS and OS in relation to haplotypes.

	Progression-free survival					Overall survival		
	Median PFS (95%CI)	HR (95% CI)	*p*	HR* (95% CI)	p*	Median OS (95%CI)	HR (95% CI)	*p*
H1/H1	13.9 (12.2–19.3)	0.73 (0.50–1.08)	0.12			28.4 (18.6–38.6)	0.86 (0.56–1.32)	
Other	11.6 (10.4–13.1)	1.00				21.1 (17.3–29.8)	1.00	0.49
H2/H2	9.59 (6.9-NA)	2.25 (1.26–4.03)	0.006	2.83 (1.33–5.99)		15.2 (9.79-NA)	1.63 (0.91–2.90)	
Other	12.52 (11.6–14)	1.00		1.00	0.006	24.4 (19.75 -30.3)	1.00	0.1
H1/H1 + H2/H2	12.3 (9.76–14.8)	0.95 (0.67–1.34)	0.75			22.6 (16.7–37.8)	1.05 (0.72–1.53)	
Other	12.0 (11.2–13.9)	1.00				23.4 (18.9–30.3)	1.00	0.8

H1/H1, Haplo1/Haplo1; H2/H2, Haplo2/Haplo2; H1/H1 + H2/H2, Haplo1/Haplo1 + Haplo2/Haplo2.

*Ajusted HR and *p*-value.

## Discussion

The present study investigated *VEGF* and *eNOS* polymorphisms in relation to clinical outcome in patients with mCRC treated with first line Bev-based chemotherapy.

The inhibition of angiogenesis, through blockade of the *VEGF*/*VEGF*R pathway, is an effective strategy in the treatment of mCRC. Positive results from randomised phase III clinical trials are available not only with Bev^9^, but also with the multikinase inhibitor regorafenib^10^ and aflibercept, a fully human fusion protein that highly specifically and potently blocks all *VEGF* isoforms and placental growth factor (PlGF)^11^. From a clinical perspective, the relatively small absolute benefit provided by these new agents, as well as the availability of an increasing number of therapeutic options, make the identification of predictive biomarkers an essential requirement for optimizing the use of antiangiogenic agents. Unfortunately, studies in this context, characterized by different designs, have shown contradictory results. In fact, although several studies have reported significant results regarding *VEGF* polymorphisms, others have failed to confirm their predictive role^12^.

For example, the retrospective study by Loupakis and colleagues hypothesized a role of the *VEGF*-1498 C/T polymorphism as a predictive biomarker of Bev efficacy, showing that patients with this genotype had a worse prognosis^13^. However, the same authors did not confirm this hypothesis in a subsequent prospective validation study^7^. In another retrospective study, *VEGF*-1498 C/T and -2578 A/C were correlated with outcome^6^. In other prospective studies, *VEGF*-1154 G/A and *VEGF*-634 G/C were significantly corre-lated with patient response to treatment^14^.

One of the most compelling reasons for the failure of predictive marker studies is the biological complexity of tumor angiogenesis. Data in the literature suggest multiple pathways in the growth of new tumor vessels, leading to study the simultaneous inhibi-tion of multiple angiogenic targets as a potentially effective strategy^15^. The role of different cell types in the development of such an intricate network of signals has also emerged and the contribution of both stromal cells and bone marrow-derived vascular progenitors recruited and stimulated by hypoxic conditions has been highlighted^16^. The relevance of the tumor microenvironment as a crucial player in the growth and stabiliza-tion of new vessels and the critical implication of the extracellular matrix in supporting angiogenesis are now well established, thus confirming the contribution of both host and tumor factors to the so called “angiogenic balance”.

Mechanisms of action of anti-*VEGF* therapy remain unclear since blockade of circu-lating *VEGF* can affect not only tumors, but also stroma and endothelial cell proliferation and maturation. While the antiangiogenic properties of Bev were initially attributed to the inhibition of endothelial cell proliferation, several biological effects are now recognized, such as the inhibition of bone marrow-derived progenitors, the normalization of vessel structure, the vascular “constriction”, the destruction of the cancer stem cell niche, the di-rect effect on cancer cells and the interaction with the host immune system. Because of these multiple mechanisms of action, the translation of in vitro and in vivo results into the human model is not immediate, and several difficulties make the development of effective preclinical models extremely complicated.

The ITACA study secondary analysis has shown that specific polymorphisms of *eNOS* were significantly correlated with patient survival and response to treatment. Nota-bly, patients with a specific combination of *eNOS* Haplo1/Haplo1 and *eNOS* Haplo2/Haplo2 had significantly longer PFS and OS and a higher ORR than those with the other genotypes^8^. The correlation was significant in the Bev-treated group and not in the chemotherapy-only group, highlighting the potential role of *eNOS* polymorphisms as pre-dictive biomarkers of Bev. Nitric oxide (NO), which is produced by the endothelium through the constitutively expressed gene known as *eNOS*, is essential for preserving the functional integrity of endothelial cells, controlling hemodynamics, and developing collateral circulation^17^. For the prevention of thrombotic and atherogenic processes, appropriate eNOS expression and activity, which results in enough NO generation, are crucial^18^. It has been demonstrated that *VEGF* inhibition causes a reduction in eNOS expression, which in turn results in a reduction in NO production^19^, and that this phenomenon is connected to the induction of hypertension, one of the most frequent dose-limiting toxicities of *VEGF* inhibitors^20^. Intron 4 *eNOS* VNTR polymorphism plays a role in regulating eNOS expression by acting as an enhancer/repressor and by coding for a 27-nt small RNA, which appears to inhibit eNOS expression at the transcriptional level^21–24^. The higher the number of 27-nt repeats, the more 27nt sir-RNA is produced, inhibiting eNOS expression. However, the association between *eNOS* VNTR in intron 4 and *eNOS* expression is still a much-debated issue^25–27^.

The purpose of the current study was to validate our previous findings, which, if verified, might have served as a reliable criterion for choosing individuals for Bev therapy.

Unfortunately, the study did not confirm the earlier findings and demonstrated the failure of both *VEGF* and *eNOS* polymorphisms as a potential predictor of benefit from Bev. The validation of haplotypes predictive role was thus compromised by the negative results on *eNOS* polymorphisms.

The lack of correlation evidence in the study could be due to the reduced power caused by fewer patients’ enrolled respect to the planned sample size. Although *eNOS* VNTR showed a significant association with ORR in univariate analysis the result was not confirmed in multivariate analysis probably because of the further reduction of sample to non-missing values.

The present study confirms the absolute importance of prospective validation as an essential step on the road of biomarkers towards clinical application. Furthermore, based on the complexity of the biology of tumor angiogenesis and the involvement of multiple actors in this process, it seems rather unlikely that a single germline SNP alone could explain the efficacy of Bev. The failure of the “candidate SNP strategy” certainly opens the way to new questions on the possibility of effectively exploiting the pharmacogenetic ap-proach to identify predictors of benefit from Bev.

This prospective study failed to validate the hypothesized predictive impact of *eNOS* and *VEGF* polymorphisms for response to Bev plus first-line chemotherapy for mCRC. The results of the previous exploratory analysis performed on patients enrolled in a ran-domized and prospective phase III multicenter study (ITACa study) were not confirmed. While on the one hand the reduction in the sample size might have played a role in the negative result, we believe that the most probable cause is biological. Furthermore, since *eNOS* is not the direct target of Bev, other factors could definitely be involved in the relationship between *eNOS* activity and Bev efficacy. Therefore the main weakness of our trial is to have hypothesized that a single polymorphism could predict such a complex event as the inhibition of angiogenesis.We believe that future directions in this research field must necessarily include more comprehensive approaches, also considering the complexity of the mechanism of angiogenesis and the mecha-nisms of action of anti-*VEGF* therapy.

## Materials and methods

### Patients and treatment

This is a prospective non-pharmacological trial aimed at validating *VEGF* and *eNOS* polymorphisms as predictors of efficacy of Bev plus first-line chemotherapy in patients with mCRC. The primary objective of the study was to assess the correlation of the selected polymorphisms with PFS. Secondary objectives were the correlations with OS and ORR. The Local Ethics Committee (Comitato Etico Area Vasta Romagna and IRST) approved the study and informed consent was obtained from all patients before blood samples were obtained for genotype testing.

This study involved 182 patients with mCRC treated with first-line chemotherapy plus Bev at the IRCCS Istituto Romagnolo per lo Studio dei Tumori (IRST) “Dino Amadori” in Meldola. Patients ≥ 18 years of age, with histologically confirmed mCRC, one or more unidimensional measurable lesions according to Response Evaluation Criteria in Solid Tumours (RECIST) 1.0, Eastern Cooperative Oncology Group (ECOG) performance status ≤ 2 (≤ 1 if age ≥ 70 years), and an estimated life expectancy of at least 12 weeks were included. Patients were required to have adequate hematological, hepatic and renal func-tion. Prior adjuvant chemotherapy for CRC or neoadjuvant/adjuvant chemo-radiotherapy for rectal cancer were permitted, if completed at least 6 months before recurrence; conversely, prior chemotherapy or immunotherapy for advanced or metastatic disease were not permitted. Patients received one of the following chemotherapy regimens in combina-tion with Bev: FOLFOX4, CAPOX, FOLFIRI, CAPIRI.

Treatment was to continue until disease progression (PD), withdrawal of consent, or unacceptable toxicity, whichever came first. If a patient became eligible for curative resection of metastatic disease, Bev was discontinued at least 6–8 weeks prior to the scheduled surgery date. After surgery, the choice of treatment was at the physician's discretion, and patients could resume treatment at least 28 days after surgery or complete wound healing, until PD. We analyzed KRAS gene (codons 12, 13, 59, 61, 117, 146), BRAF gene (codons 594, 600, 601) and NRAS gene (codons 12, 13, 18, 59, 61, 117, 146) as for clinical practice.

### Polymorphisms and genotyping

*VEGF* SNP (*VEGF* + 936C > T), *eNOS* SNP (*eNOS* + 894G > T) and a 27-nucleotide variable number tandem repeat (VNTR) of *eNOS* were analyzed.

*VEGF* + 936C > T (re3025039) is located in 3’UTR region, *eNOS* + 894G > T (rs1799983) is located within exon 7, while *eNOS* VNTR 27 bp 4a/b is located in intron 4 of the gene. *eNOS* VNTR 27 bp 4a/b has 2 common alleles: “4°” with 4 repeats of 27 nucleotides and “4b” with 5 repeats.

The analysis of the selected polymorphisms was performed on peripheral blood samples. Genomic DNA was extracted from 200 μl of whole blood using the QIAamp DNA Mini Kit (Qiagen SPA, Milan, Italy) and quantified with the Nanodrop instrument (Celbio Spa, Milan, Italy).

*eNOS* + 894 were analyzed by Real time PCR (7500 Applied Biosystems) with TaqMan SNP Genotyping assays (Assay ID C_3219460_20) (Applied Biosystems, Foster City, CA, USA) and starting from 10 ng of DNA. The *eNOS* VNTR and *VEGF* + 936 polymorphisms were instead determined by standard PCR and direct sequencing analysis on the ABI 3130 sequencer (Applied Biosystems). PCRs were performed starting from 50 ng of genomic DNA. PCR conditions and primer sequences for *VEGF* + 936 and *eNOS* VNTR were report-ed in our previous study^8^.

All samples were analyzed at the Biosciences Laboratory of IRST IRCCS (Meldola, FC).

### Statistical analysis

Based on the results obtained from the ITACa study, we assumed a prevalence of the haplotype to be validated (homozygosity in *eNOS* Haplo 1 and homozygosity in *eNOS* Haplo2, Haplo1: 4b-G, Haplo2: 4b-T based on *eNOS* VNTR 4a/b and *eNOS* polymor-phisms + 894 G/T) of 0.5. By establishing a significance threshold of 5%, a type II error (2-tailed) of 20%, a median PFS of 9 months for the haplotype other than Hap-lo1/Haplo1 + Haplo2/Haplo2, and a hazard ratio (HR) of 0.65, we estimated a total of 200 patients to be recruited.

Patient characteristics were summarized using the median and range for the contin-uous variables and frequencies and percentages for the categorical variables. PFS was de-fined as the time between the date of diagnosis and that of progression or death; OS was defined as the time between the date of diagnosis and death; ORR was defined as the per-centage of patients achieving a complete (CR) or partial response (PR).

The Kaplan–Meier method was used to estimate the time-to-event curves and the log-rank test was applied for their comparison. HRs for each polymorphism and haplo-types and relative 95% CI were calculated using the Cox proportional hazards model. The association between polymorphisms or haplotypes and ORR was analyzed by a logistic regression model and the effect of polymorphisms on ORR was estimated by odds ratio (OR) and 95% CI.

Multivariate analyses were performed after univariate analyses revealed a significant effect of the polymorphism/haplotype. Models were adjusted for chemotherapy regimen (FOLFOX/CAPOX vs FOLFIRI/CAPIRI vs other), gender, age, *KRAS* status, *NRAS* status, *BRAF* status, tumour location (rectum vs colon) as probable confounders and prognostic factors^8^.

All *p*-values were based on two-sided testing and *p* < 0.05 were considered statistically significant. Analyses were performed using R (version 4.1.0).

### Ethical approval

The study was conducted according to the guidelines of the Declaration of Helsinki, and approved by the Institutional Ethics Committee of IRCCS Istituto Romagnolo per lo Studio dei Tumori (IRST) “Dino Amadori”, Meldola, Italy (reference number IRSTB038 and date of approval 08/07/2015).

### Consent for publication

Informed consent was obtained from all subjects involved in the study. Written informed consent has been obtained from the patients to publish this paper.

### Supplementary Information

Supplementary Information 1.

Supplementary Information 2.

## Data Availability

The data supporting the fndings of this study could be obtained from the corresponding author.
