# Supplementary material for: Prospective validation of VEGF and eNOS polymorphisms as predictors of first-line bevacizumab efficacy in patients with metastatic colorectal cancer
Source: Sci Rep. 2023 Aug 9;13:12921. doi: 10.1038/s41598-023-40220-7 (PMC10412588; doi:10.1038/s41598-023-40220-7)
Supplement: Supplementary file 1 — Supplementary Information 1. [file 41598_2023_40220_MOESM1_ESM.pptx]

## Slide 1
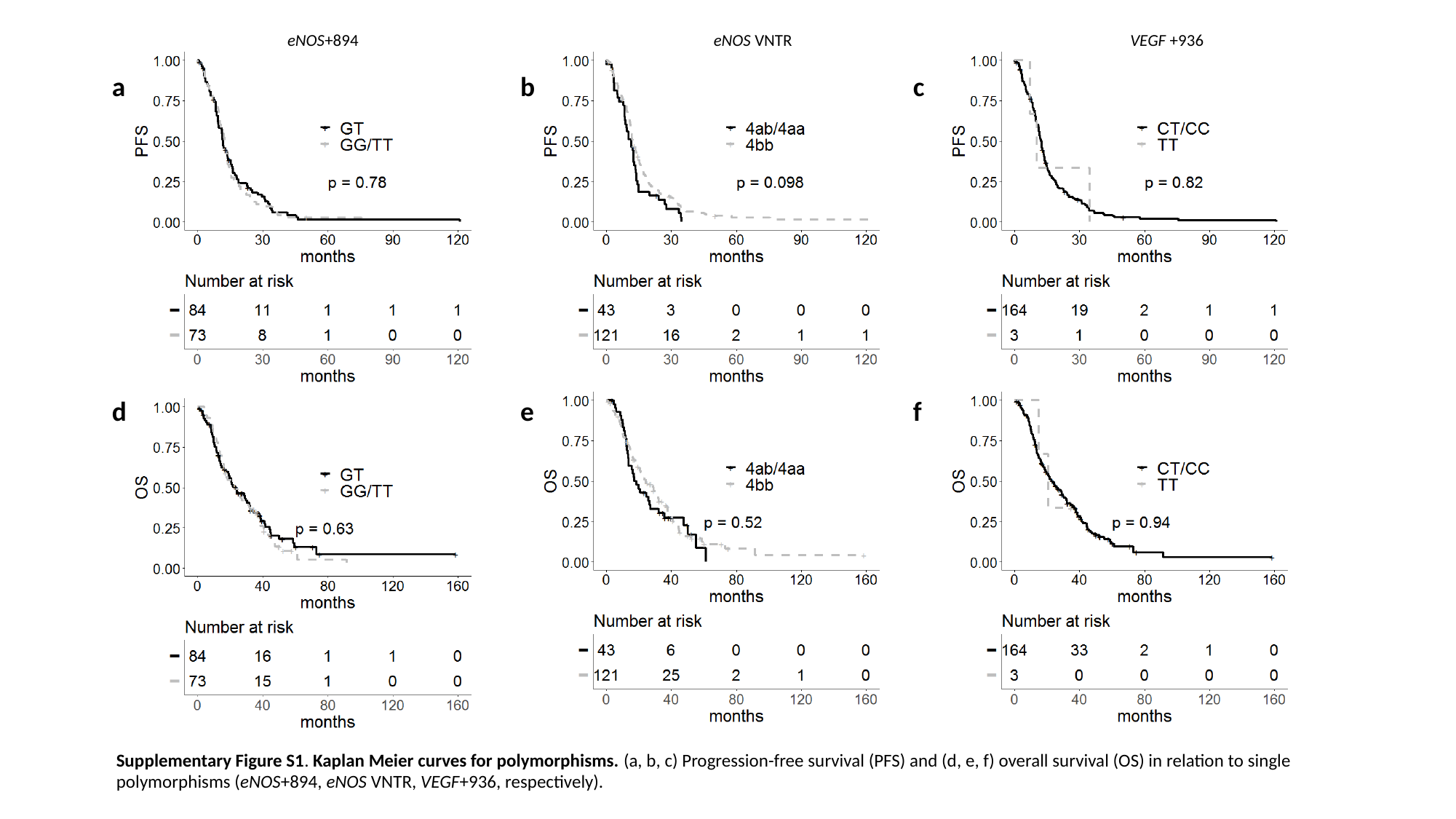

eNOS+894
eNOS VNTR
VEGF +936
a
b
c
d
e
f
Supplementary Figure S1. Kaplan Meier curves for polymorphisms. (a, b, c) Progression-free survival (PFS) and (d, e, f) overall survival (OS) in relation to single polymorphisms (eNOS+894, eNOS VNTR, VEGF+936, respectively).
