# Supplementary material for: Prospective validation of VEGF and eNOS polymorphisms as predictors of first-line bevacizumab efficacy in patients with metastatic colorectal cancer
Source: Sci Rep. 2023 Aug 9;13:12921. doi: 10.1038/s41598-023-40220-7 (PMC10412588; doi:10.1038/s41598-023-40220-7)
Supplement: Supplementary file 2 — Supplementary Information 2. [file 41598_2023_40220_MOESM2_ESM.docx]

**Supplementary Information**

**Supplementary Fig. S1**. Kaplan Meir curves for polymorphisms. (a, b, c) Progression-free survival (PFS) and (d, e, f) overall survival (OS) in relation to single polymorphisms (*eNOS*+894, *eNOS* VNTR, *VEGF*+936, respectively).

**Supplementary Table S1**. Polymorphisms and objective response rate

**Supplementary Table S2**. Haplotype and objective response rate.

**Supplementary Table S1.** Polymorphisms and objective response rate

|  |  |  |  | **Univariate analysis** | | **Multivariate analysis** | |
| --- | --- | --- | --- | --- | --- | --- | --- |
|  | **No. of patients** | **CR/PR (%)** | **SD/PD (%)** | **OR (95% CI)** | **P** | **OR* (95% CI)** | **P*** |
| ***eNOS*+894G>T** |  |  |  |  |  |  |  |
| GT | 57 | 41 (2%) | 16 (28%) | 0.90 (0.38-2.12) | 0.81 |  |  |
| GG/TT | 50 | 37 (74%) | 13 (26 %) | 1.00 |  |  |  |
| ***eNOS* VNTR** |  |  |  |  |  |  |  |
| 4bb | 77 | 61 (79%) | 16 (21%) | 2.81 (1.16-6.79) | **0.02** | 2.46 (0.85-7.10) | 0.82 |
| 4ab/4aa | 33 | 19 (58%) | 14 (42%) | 1.00 |  | 1.00 |  |
| ***VEGF* +936** |  |  |  |  |  |  |  |
| TT | 112 | 81 (72%) | 31 (28%) |  |  |  |  |
| CT/CC | 2 | 2 (100%) | 0 |  |  |  |  |

***** Adjusted for CT (FOLFOX/CAPOX vs FOLFIRI/CAPIRI vs Other), gender, age, *KRAS* status, *NRAS* status, *BRAF* status, tumor localization (rectum vs colon).

Abbreviations: CR, complete response; PR, partial response; SD, stable disease; PD, progression disease; OR, odds ratio; CI, confidence interval.

**Supplementary Table S2.** Haplotype and objective response rate

|  | **No. of patients** | **CR/PR (%)** | **SD/PD (%)** | **OR (95% CI)** | **P** |
| --- | --- | --- | --- | --- | --- |
| Haplo 1/Haplo 1 | 19 | 16 (84%) | 3 (16%) | 2.26 (0.60-8.45) | 0.23 |
| Other | 84 | 59 (70%) | 25 (30%) | 1.00 |  |
| Haplo 2/Haplo 2 | 12 | 9 (75%) | 3 (25%) | 1.14 (0.28-4.54) | 0.86 |
| Other | 91 | 66 (73%) | 25 (27%) | 1.00 |  |
| Haplo 1/Haplo 1 +  Haplo2/ Haplo2 | 31 | 25 (81%) | 6 (19%) | 1.83 (0.66-5.10) | 0.25 |
| Other | 72 | 50 (69%) | 22 (31%) | 1.00 |  |

Abbreviations: CR, complete response; PR, partial response; SD, stable disease; PD, progression disease; OR, odds ratio; CI, confidence interval.
